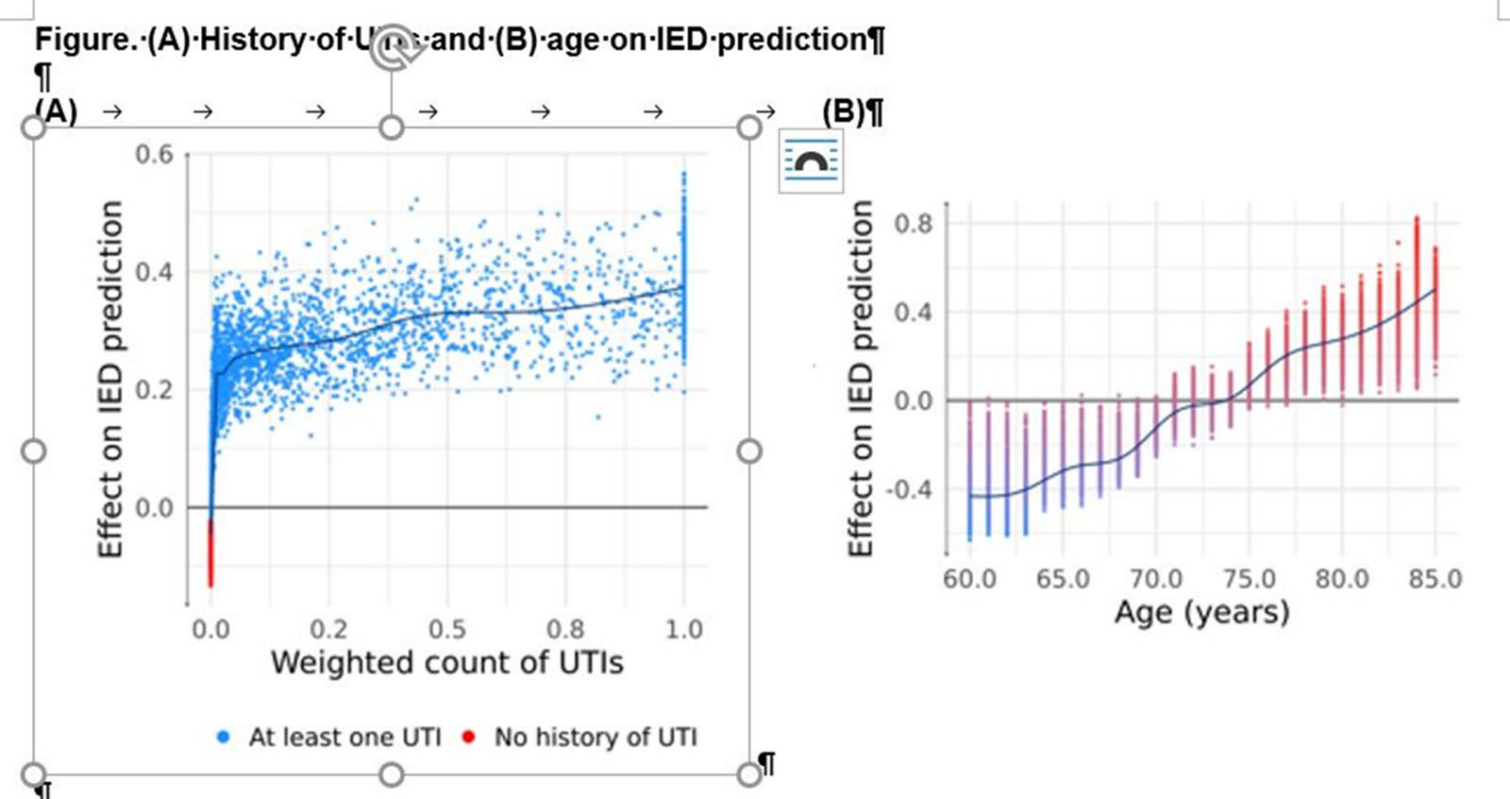# Identification of Risk Factors for Invasive Extraintestinal Pathogenic *Escherichia coli* (ExPEC) Disease

**DOI:** 10.1017/ash.2021.40

**Published:** 2021-07-29

**Authors:** Erik Clarke, Jeroen Geurtsen, Bart Spiessens, Christel Chehoud

## Abstract

**Background:** A pathogenic group of invasive extraintestinal pathogenic (ExPEC) *Escherichia coli* possess the ability to infect normally sterile body sites and cause severe invasive ExPEC disease (IED). ExPEC is a leading cause of bacteremia and sepsis worldwide and is associated with older age and multidrug-resistant infections. Janssen Vaccines & Prevention is developing a novel multivalent glycoconjugate vaccine to prevent IED. We aimed to use an unbiased approach, with no prespecified potential risk factors, using machine-learning models, to screen for and identify IED risk factors for further validation. **Methods:** We used a patient-level prediction study design to model the probability of a patient developing IED within 14 days to 1 year from a given date based on their prior 2 years of health records. We used the Optum EHR database (~98 million subjects) in the common data model (CDM) format, with health features encoded in the following categories: conditions, procedures, drugs, healthcare visits, recent laboratory measurements, and age and gender. A gradient boosting model (XGBoost) was used with Shapley additive explanation (SHAP) values to identify which features were most important to the model’s decisions and to characterize precisely the relationship between features and outcomes (binary or continuous). **Results:** Study participants were aged ≥60 years at index with no previously recorded IED. Of ~6,500,000 cases included, ~8,000 had IED during the prediction window. We found that having ≥1 urinary tract infection (UTI) in the retrospective period increased the model’s probability of predicting IED for that patient, with more frequent or more recent UTIs increasing IED prediction chance (Figure 1). Higher age linearly increased the model’s likelihood of predicting that a patient would develop IED. The model also identified ≥1 inpatient or ER visit and laboratory values indicative of renal or immune dysfunction to be correlated with increased IED risk. This methodology is a generalizable approach to screening for potential risk factors for an outcome using EHR databases; it requires little to no prespecification of the health factors or precise relationship between the factors and outcome. **Conclusions:** Using a new, impartial methodology (with no prespecification), older age and a history of UTIs were key predictive features for IED, factors previously identified through traditional analysis, confirming the validity of the methodology. Novel features, including recent hospitalization, were shown to increase IED risk relative to existing criteria. Our findings may be used to inform the clinical development of preventive strategies.

**Funding:** Janssen Research and Development

**Disclosures:** None

**Figure 1.**